# Hsp90 Levels in Idiopathic Inflammatory Myopathies and Their Association With Muscle Involvement and Disease Activity: A Cross-Sectional and Longitudinal Study

**DOI:** 10.3389/fimmu.2022.811045

**Published:** 2022-01-28

**Authors:** Hana Štorkánová, Sabína Oreská, Maja Špiritović, Barbora Heřmánková, Kristýna Bubová, Olga Kryštůfková, Heřman Mann, Martin Komarc, Kryštof Slabý, Karel Pavelka, Ladislav Šenolt, Josef Zámečník, Jiří Vencovský, Michal Tomčík

**Affiliations:** ^1^ Institute of Rheumatology, Prague, Czechia; ^2^ Department of Rheumatology, 1^st^ Faculty of Medicine, Charles University, Prague, Czechia; ^3^ Department of Physiotherapy, Faculty of Physical Education and Sport, Charles University, Prague, Czechia; ^4^ Department of Methodology, Faculty of Physical Education and Sport, Charles University, Prague, Czechia; ^5^ Department of Rehabilitation and Sports Medicine, 2^nd^ Faculty of Medicine, Charles University and University Hospital Motol, Prague, Czechia; ^6^ Department of Pathology and Molecular Medicine, 2^nd^ Faculty of Medicine, Charles University and University Hospital Motol, Prague, Czechia

**Keywords:** heat shock protein 90, idiopathic inflammatory myopathies, skeletal muscle involvement, disease activity, disease damage, response to treatment

## Abstract

**Background:**

Heat shock proteins (Hsp) are chaperones playing essential roles in skeletal muscle physiology, adaptation to exercise or stress, and activation of inflammatory cells. We aimed to assess Hsp90 in patients with idiopathic inflammatory myopathies (IIM) and its association with IIM-related features.

**Methods:**

Hsp90 plasma levels were analyzed in a cross-sectional cohort (277 IIM patients and 157 healthy controls [HC]) and two longitudinal cohorts to assess the effect of standard-of-care pharmacotherapy (n=39 in early disease and n=23 in established disease). Hsp90 and selected cytokines/chemokines were measured by commercially available ELISA and human Cytokine 27-plex Assay.

**Results:**

Hsp90 plasma levels were increased in IIM patients compared to HC (median [IQR]: 20.2 [14.3–40.1] vs 9.8 [7.5–13.8] ng/mL, p<0.0001). Elevated Hsp90 was found in IIM patients with pulmonary, cardiac, esophageal, and skeletal muscle involvement, with higher disease activity or damage, and with elevated muscle enzymes and crucial cytokines/chemokines involved in the pathogenesis of myositis (p<0.05 for all). Plasma Hsp90 decreased upon pharmacological treatment in both patients with early and established disease. Notably, Hsp90 plasma levels were slightly superior to traditional biomarkers, such as C-reactive protein and creatine kinase, in differentiating IIM from HC, and IIM patients with cardiac involvement and interstitial lung disease from those without these manifestations.

**Conclusions:**

Hsp90 is increased systemically in patients with IIM. Plasma Hsp90 could become an attractive soluble biomarker of disease activity and damage and a potential predictor of treatment response in IIM.

## Introduction

Idiopathic inflammatory myopathies (IIM) are a heterogeneous group of rare acquired diseases characterized by inflammatory, immune-mediated skeletal muscle involvement associated with mostly painless, symmetric, and predominantly proximal muscle weakness and low muscle endurance. In addition, IIM may also affect the skin and internal organs, mostly the lungs, esophagus, and heart (1). Based on specific clinical and immunological features and histopathology of the skeletal muscle, IIM can be classified into four major subtypes: polymyositis (PM), dermatomyositis (DM), inclusion body myositis (IBM), and immune-mediated necrotizing myopathy (IMNM) (2). Although the exact mechanisms of pathogenesis are still ill-defined, these complex autoimmune disorders are believed to develop as a consequence of abnormal immune activation and non-immune mechanisms, such as autophagy dysregulation, the presence of free radicals, and endoplasmic reticulum (ER) stress (3). ER stress leads to non-immune mediated muscle damage, accumulated unfolded proteins, and imbalanced muscular protein homeostasis (3, 4). Processes caused by ER stress activate heat-shock protein (Hsp) response to protect muscle cells against protein accumulation and inflammation (3, 5).

Heat shock proteins belong to a large family of molecular chaperones, which were discovered upon exposing cells to higher temperatures. Their primary role is to stabilize, activate, and protect proteins against degradation in the proteasome (6). Hsp90 is a highly ubiquitous ATP-dependent molecular chaperone, which is involved in several biological processes, e.g., controlling cell cycle and survival, maintaining homeostasis, stabilizing and activating cellular proteins. It plays an important role in the innate and adaptive immune system through activation and maturation of antigen-presenting cells and induction of pro-inflammatory cytokines [e.g., interleukin (IL)-1β, IL-12, and tumor necrosis factor (TNF)] (6, 7). Hsp90 also participates in autoimmune response, oncogenesis, viral infections, and neurodegenerative diseases (7–10).

To date, only a few studies examined the role of Hsp90 in rheumatic diseases. In rheumatoid arthritis (RA), an association of serum Hsp90 with disease activity and systemic inflammation was found despite the lack of significant differences between patients and healthy controls (11). Similarly, increased Hsp90 expression in peripheral blood mononuclear cells was detected in patients with more active systemic lupus erythematosus (SLE) (12). Our previous study described an increased expression of Hsp90 in the involved skin of scleroderma (SSc) patients and demonstrated its pro-fibrotic properties both *in vitro* and *in vivo* (13). Recently, we demonstrated a potential therapeutic application of Hsp90 inhibitors in SSc and showed increased Hsp90 plasma levels in SSc patients, predominantly in patients with skin and lung involvement (14, 15). In axial spondyloarthritis (ax-SpA), we observed elevated Hsp90 plasma levels, which were associated with inflammation of the sacroiliac joints (16). A potential role of Hsp90 in IIM was investigated by De Paepe et al., who described an increased local expression of Hsp90 in muscle biopsy samples from patients with PM, DM, and IBM, specifically in the regenerating and atrophic muscle fibers, and in macrophages and cytotoxic T-cells actively invading non-necrotic muscle fibers. In spite of the potentially protective effect of Hsp90 expression in regenerating muscle fibers, more evidence suggests the negative and pro-inflammatory role of Hsp90 in IIM (17–19).

Given the lack of organ- and disease-specific biomarkers for IIM, we aimed to examine the systemic levels of Hsp90 in patients with IIM and in healthy individuals, to analyze their association with IIM-related clinical features, and to evaluate their response to pharmacological intervention.

## Materials and Methods

### Patients and Healthy Controls

For the cross-sectional analysis of systemic levels of Hsp90, plasma samples were obtained from 277 Caucasian patients with IIM and 157 healthy Caucasian individuals. In total, two longitudinal cohorts (LC) were included to assess the effect of standard-of-care pharmacological therapy (LC1, LC2). LC1 included consecutively recruited IIM patients from the cross-sectional cohort with a short disease duration (≤ 6 months since the first symptom). Additionally, patients in LC1 had plasma samples and clinical data available at the 6-month and 12-month follow-ups after treatment initiation. LC2 was comprised of consecutively recruited IIM patients with established disease and ongoing treatment. Patients in LC2 had plasma samples and clinical data available at the 6-month follow-up after treatment initiation, and were originally recruited as a control group (i.e., treated with a standard-of-care pharmacotherapy only) in our recently published study on physiotherapy intervention in IIM patients (20). Patients with DM and PM were diagnosed using the Bohan and Peter classification criteria (21), and patients with IMNM fulfilled the definite or probable criteria of the European Neuromuscular Centre (ENMC) (22). Patients with dermatomyositis who developed cancer within three years of the diagnosis were classified as CDM (23). General age-appropriate screening was performed in all IIM patients; it was guided by clinical suspicion and included basic noninvasive examinations, such as chest X-ray, ultrasound of the abdomen, fecal occult blood, and gynecological evaluation or prostate-specific antigen analysis. For patients with DM and positivity of anti-TIF1-γ, anti-NXP2, or anti-SAE, and for patients without other myositis-specific antibodies (MSAs) or myositis-associated antibodies (MAAs), an additional extensive malignancy screening was performed, including serum cancer markers, comprehensive blood tests, whole-body PET-CT or CT scans of the chest, abdomen, and pelvis.

### Clinical Evaluation

All patients have been clinically evaluated and treated at the Institute of rheumatology in Prague (IoRP) from 2007 by a board-certified rheumatologist experienced in diagnosing and treating IIM (JV, HM, OK) according to international guidelines (24). Physical functioning and clinical disease activity were assessed by the core set measures of disease activity proposed by the International Myositis Assessment & Clinical Studies Group (IMACS): myositis disease activity assessment tool (MDAAT), which includes the assessment of muscle disease activity (MDA) and physician global assessment of disease activity using the visual analogue scale (VAS), manual muscle testing (MMT) and the health assessment questionnaire (HAQ) (25). In addition to MMT8, we routinely measured muscle strength also in m. triceps brachii and m. iliopsoas. The myositis damage index (MDI) was used to evaluate IIM associated damage (25). All participants gave informed consent prior to participation. Our research was approved by the Ethics Committee of the IoRP. All methods were performed in accordance with the relevant guidelines and regulations.

### Laboratory Measurements

Peripheral blood samples were collected into commercially available EDTA-treated tubes, immediately centrifuged, and stored at -80°C until use. Serum levels of C-reactive protein (CRP), creatine phosphokinase (CK), lactate dehydrogenase (LD), alanine aminotransferase (ALT), aspartate aminotransferase (AST), and myoglobin (Mb) were determined using Beckman CoulterAU 680 analyzer (Beckman Coulter, USA). Erythrocyte sedimentation rate (ESR) was measured according to the Fahreus and Westergren method. Antinuclear antibodies (ANA) were detected using indirect immunofluorescence on HEP2 cells (ImmunoConcepts, Sacramento, CA, USA), and the MSAs and MAAs were determined by Myositis Line Immunoassay (IMTEC, Berlin, Germany) (detects anti-Mi-2, anti-PM-Scl, anti-Jo-1, anti-PL-7, anti-PL-12 antibodies) and Myositis Westernblot (Euroimmun, Lübeck, Germany) (detects anti-Mi-2, anti-TIF1-γ, anti-MDA5, anti-NXP2, anti-SAE, anti-Jo-1, anti-SRP, anti-PL-7, anti-PL-12, anti-EJ, anti-OJ antibodies). Plasma Hsp90 was quantified using a commercially available high-sensitive ELISA kit (eBioscience, Vienna, Austria) according to the manufacturer’s protocol. The calculated sensitivity is 0.03 ng/mL, and the absorbance value was determined at 450 nm by an ELISA reader (SUNRISE; Tecan, Grödig, Austria). The plasma concentrations of selected cytokines/chemokines were measured by a commercially available Bio-Plex Pro™ human Cytokine 27-plex Assay (BIO-RAD, California, USA) according to the manufacturer’s instruction. This kit measures the concentration of 27 cytokines, chemokines or growth factors: interleukin (IL)-1β, IL-1RA, IL-2, IL-4, IL-5, IL-6, IL-7, IL-8, IL-9, IL-10, IL-12 (p70), IL-13, IL-15, IL-17A, eotaxin, fibroblast growth factor (FGF) basic, granulocyte colony-stimulating factor (G-CSF), granulocyte-macrophage colony-stimulating factor (GM-CSF), interferon-γ (IFN-γ), interferon gamma-induced protein (IP)-10, monocyte chemoattractant protein (MCP)-1 (CCL2), macrophage inflammatory proteins (MIP)-1α (CCL3), MIP-1β (CCL4), platelet-derived growth factor (PDGF)-bb, regulated on activation/normal T cell expressed and secreted (RANTES, CCL5), tumor necrosis factor (TNF), and vascular endothelial growth factor (VEGF). The absorbance of the Bio-Plex Pro™ human Cytokine 27-plex Assay (BIO-RAD, California, USA) was established by Luminex BIO-PLEX 200 System (Bio-Rad, California, USA). Samples were measured as duplicates and the mean value was used.

### Statistical Analysis

Basic descriptive statistics (mean, median, interquartile range [IQR], skewness, and kurtosis) were computed for all variables, which were subsequently tested for normality using the Kolmogorov–Smirnov and Shapiro-Wilk tests. Differences in interval variables (e.g., Hsp90) were evaluated using the Mann-Whitney U test, while the chi-square test was used to compare frequency counts of categorical variables (e.g., gender). The bivariate relationships between variables under study were assessed using the Spearman correlation coefficient. A correlation coefficient (r) of 0.1 to 0.3 was considered weak, 0.3 to 0.5 was considered moderate, and 0.5 to 1.0 was considered strong. Multivariate regression analysis was used to predict patients’ Hsp90 levels by a set of predictors (levels of muscle enzymes, MITAX, and current prednisone equivalent dose). Repeated longitudinal measurements were analyzed by one-way repeated ANOVA followed by *post hoc* comparisons. Diagnostic utility of Hsp90 plasma levels was assessed by the receiver operating characteristic (ROC) curve and area under the curve (AUC) analyses. Data are presented as median (IQR) unless stated otherwise. Statistical significance was set at p<0.05. All analyses were conducted using SPSS version 25 (SPSS, Inc., Chicago, IL, USA). Graphs were created using GraphPad Prism 5 (version 5.02; GraphPad Software, La Jolla, CA, USA).

## Results

### Characteristics of Patients

The cross-sectional study group comprised 104 patients with PM, 104 patients with DM, 42 patients with cancer-associated dermatomyositis (CDM), and 27 patients with IMNM. The clinical and demographic characteristics of this cohort are shown in **Table 1**. The median interval between the diagnosis of cancer and the subsequent diagnosis of DM (n=19) was 24 months, whereas the median interval between the diagnosis of DM and the subsequent confirmation of cancer (n=23) was 3 months. The most prevalent malignancies involved the breast (29%), ovaries (17%), lung (12%), kidney (10%), thymus (10%), and esophagus, urinary bladder, and liver (5% each). Cancer of the cervix, lymph nodes, skin, and large intestines was present in one patient each. The most prevalent antibodies in CDM patients included anti-TIF1-γ (43%), anti-Ro-52 (17%), anti-Mi-2 (12%), and anti-PM-Scl (5%). Whereas anti-NXP2, anti-Jo-1, and anti-Ro-60 were present only in one patient each; 7 patients (17%) had no detectable MSAs or MAAs.

**Table 1:** Clinical and demographic characteristics of IIM patients and healthy controls: cross-sectional cohort.

Parameters	IIM patients (n=277)	Healthy controls (n=157)	p-value
Sex: Female/Male, n (%)	198 (71)/79 (29)	92 (59)/65 (41)	0.006
Age, years	56.5 (45.9 – 64.4)	46.0 (34.0 – 60.0)	0.001
**Clinical features**			
Disease duration, years	1.7 (0.6 – 5.9)		
			
IIM subtype, n (%): PM/DM/	104 (37.5)/104 (37.5)/		
CDM/IMNM	42 (15)/27 (10)		
IIM-associated symptoms, n (%):			
MW/SR/MH/RP/	236 (85)/133 (48)/96 (35)/78 (28)/		
A/ILD/CI/D	76 (27)/126 (45)/50 (18)/120 (43)		
MITAX	0.13 (0.08 – 0.21)		
MYOACT	0.05 (0.02 – 0.12)		
MDI extent	0.08 (0.03 – 0.14)		
MDI severity	0.03 (0.01 – 0.06)		
MDI extended	0.08 (0.00 – 0.13)		
MMT-8	65.0 (55.0 – 74.0)		
HAQ	0.9 (0.3 – 1.5)		
**Laboratory features**			
Autoantibodies, n (%):			
ANA/Ro-52/Jo-1/TIF1/PM-Scl/	124 (45)/80 (29)/64 (23)/21 (8)/29 (11)/		
Mi-2/Ro-60/HMGCR/SRP/U1RNP/MDA5/	20 (7)/18 (6.5)/14 (5)/10 (4)/9 (3)/6 (2.2)/		
La/PL-7/Ku/SAE/NXP2	4 (1.4)/3 (1.1)/2 (0.7)/2 (0.7)/1 (0.4)		
CRP, mg/L	2.5 (1.3 – 6.3)		
CK, μkat/L	2.6 (0.9 – 12.4)		
LD, μkat/L	4.8 (3.5 – 7.4)		
**Current treatment**			
Prednisone equivalent dose, mg/day	20.0 (7.5 – 47.5)		
GC/MTX/AZA/	227 (82)/83 (30)/22 (8)/		
CPA/CSA, n (%)	1 (0.3)/5 (2)		

Data are presented as median (inter-quartile range) unless stated otherwise; A, arthritis; ANA, antinuclear antibodies; AZA, azathioprine; CDM, cancer-associated dermatomyositis; CI, cardiac involvement; CK, creatine kinase; CPA, cyclophosphamide; CRP, C-reactive protein; CSA, cyclosporin A; D, dysphagia; DM, dermatomyositis; GC, glucocorticoids; HAQ, Health Assessment Questionnaire; HMGCR, anti-3-hydroxy-3-methylglutaryl-CoA reductase; IIM, idiopathic inflammatory myopathy; ILD, interstitial lung disease; IMNM, immune-mediated necrotizing myopathy; Jo-1, anti-histidyl-tRNA synthetase; Ku, anti-Ku (against the nuclear DNA-dependent protein kinase subunit); La, anti-La (against La(SS-B), a nuclear 47 kD phosphoprotein, associated with small RNA synthesized by RNA polymerase III); LD, lactate dehydrogenase; MDA5, anti-CADM-140 (melanoma differentiation-associated gene 5); MDI, Myositis Damage Index; MH, mechanic’s hands; Mi-2, antinuclear helicase 218/240 kDa; MITAX, Myositis Intention to Treat Activity Index; MMT-8, Manual Muscle Testing of eight muscles; MTX, methotrexate; MW, muscle weakness; MYOACT, Myositis Disease Activity Assessment visual analogue scale; NXP2, anti-NXP2 (nuclear matrix protein); PL-7, anti-threonyl-tRNA synthetase; PM, polymyositis; PM-Scl, anti-Pm-Scl (anti-core complex 11-16 proteins); Ro, anti-Ro (52/60kDa, against cytoplasmic RNA and associated peptides); RP, Raynaud’s phenomenon; SAE, anti-SUMO1 (small ubiquitin-like modifier 1) activating enzyme; SR, skin rash; SRP, anti-signal recognition particles; TIF1, anti-TIF1 (transcriptional intermediary factor-1); U1RNP, anti-U1-RNP (ribonucleoprotein).

The first longitudinal cohort (LC1) comprised 39 Caucasian IIM patients from the cross-sectional cohort, including 11 treatment-naïve patients and 28 patients with a short disease duration (median disease duration: 1.2 months), who recently started pharmacological treatment. The clinical and demographic characteristics of this cohort are shown in **Table 2**.

**Table 2:** Baseline clinical characteristics of treatment-naïve and early-disease IIM patients treated with standard-of-care pharmacological therapy: longitudinal cohort 1.

Parameters	Baseline (Month 0) (n=39)	Month 6 (n=39)	Month 12 (n=39)
Sex: Female/Male, n (%)	29 (74)/10 (26)		
Age, years	57.1 (53.2 – 63.3)		
**Clinical features**			
Disease duration, months	1.2 (0.0 – 2.8)		
IIM subtype, n (%): PM/DM/	13 (33)/18 (46)/		
IMNM	8 (21)		
IIM-associated symptoms, n (%): MW/SR/MH/	39 (100)/24 (62)/14 (36)/		
RP/A/ILD/	10 (26)/10 (26)/26 (67)/		
CI/D	8 (21)/21 (54)		
MITAX	0.2 (0.1 – 0.3)		
MYOACT	0.1 (0.03 – 0.2)		
MDI extent	0.11 (0.03 – 0.13)		
MMT-8	65.0 (55.0 – 71.0)		
HAQ	0.9 (0.4 – 1.4)		
**Laboratory features**			
Autoantibodies, n (%): ANA/Ro-52/Jo-1/TIF1/	15 (38)/11 (28)/11 (28)/1 (3)/		
PM-Scl/Mi-2/HMGCR/Ro-60/SRP/U1RNP	4 (10)/4 (10)/4 (10)/3(7)//2 (6)/1 (3)		
CRP, mg/L	2.7 (1.3 – 8.1)	2.2 (1.0 – 8.6)	1.6 (0.9 – 15.0)
CK, μkat/L	3.8 (1.3 – 18.3)	1.4 (0.6 – 7.3)	1.4 (0.7 – 3.7)
LD, μkat/L	5.9 (4.0 – 8.5)	4.0 (3.5 – 6.2)	4.1 (3.0 – 4.9)
**Current treatment:**			
Prednisone equivalent dose, mg/day	20 (0 – 50)	20 (10 – 25)	10 (7.5 – 20)
MTX/AZA, n (%)	9 (23)/3 (8)	14 (36)/5 (13)	12 (31)/9 (23)

Data are presented as median (inter-quartile range) unless stated otherwise; A, arthritis; ANA, antinuclear antibodies; AZA, azathioprine; CDM, cancer-associated dermatomyositis; CI, cardiac involvement; CK, creatine kinase; CRP, C-reactive protein; D, dysphagia; DM, dermatomyositis; HAQ, Health Assessment Questionnaire; HMGCR, anti-3-hydroxy-3-methylglutaryl-CoA reductase; IIM, idiopathic inflammatory myopathy; ILD, interstitial lung disease; IMNM, immune-mediated necrotizing myopathy; Jo-1, anti-histidyl-tRNA synthetase; LD, lactate dehydrogenase; MDI, Myositis Damage Index; MH, mechanic’s hands; Mi-2, antinuclear helicase 218/240 kDa; MITAX, Myositis Intention to Treat Activity Index; MMT-8, Manual Muscle Testing of eight muscles; MTX, methotrexate; MW, muscle weakness; MYOACT, Myositis Disease Activity Assessment visual analogue scale; PM, polymyositis; PM-Scl, anti-Pm-Scl (anti-core complex 11-16 proteins); Ro, anti-Ro (52/60kDa, against cytoplasmic RNA and associated peptides); RP, Raynaud’s phenomenon; SR, skin rash; SRP, anti-signal recognition particles; TIF1, anti-TIF1 (transcription intermediary factor-1); U1RNP, anti-u1-rnp (ribonucleoprotein).

The second longitudinal cohort (LC2) comprised 23 Caucasian IIM patients with established disease (median disease duration: 2.8 years) and ongoing pharmacological treatment. The clinical and demographic characteristics of this cohort are shown in **Supplementary Table 1**.

### Plasma Hsp90 in Patients With Idiopathic Inflammatory Myopathies and in Healthy Controls: A Cross-Sectional Analysis

**Figure 1:**
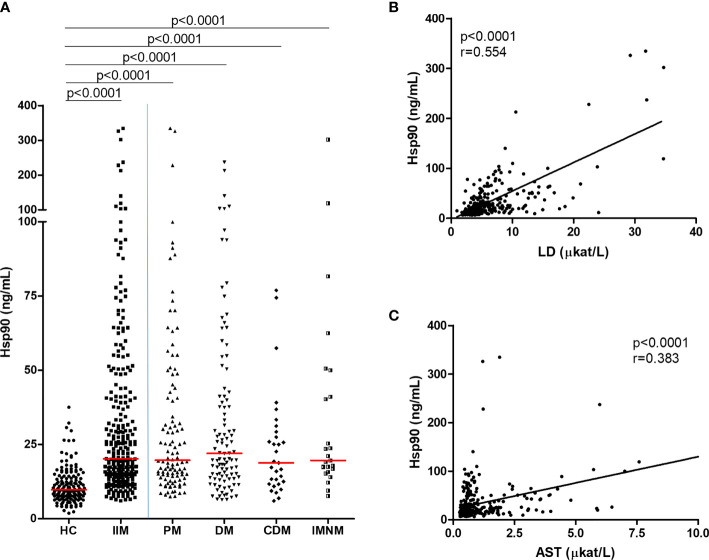
Plasma Hsp90 levels were increased in patients with IIM compared to healthy controls and were associated with serum markers of muscle damage. **(A)** Systemic levels of Hsp90 are significantly elevated in patients with idiopathic inflammatory myopathies (IIM) and individual subtypes of IIM (PM, polymyositis; DM, dermatomyositis; CDM, cancer-associated dermatomyositis and IMNM, immune-mediated necrotizing myopathy) compared to healthy controls (HC). Horizontal bars represent the median. Increased plasma Hsp90 is associated with elevated serum levels of **(B)** lactate dehydrogenase (LD) and **(C)** aspartate aminotransferase (AST).

We observed increased plasma levels of Hsp90 in all IIM patients (n=277) compared to healthy individuals (n=157) (median [inter-quartile range]: 20.2 [14.3–40.1] vs 9.8 [7.5–13.8] ng/mL, p<0.0001). Circulating Hsp90 levels were also elevated in all individual subgroups of IIM compared to healthy controls (PM: 19.7 [14.3–42.2], DM: 22.0 [14.1–41.2], CDM: 18.9 [11.7–29.7], IMNM: 19.6 [16.3–45.5] ng/mL, p<0.0001 for all comparisons) (**Figure 1A**). Since healthy controls were significantly younger with a higher proportion of males compared to our IIM cohort (**Table 1**), additional analysis was performed, and all above-mentioned differences remained statistically significant (p<0.001 for all) even after adjusting for age and sex (using a general linear model). Interestingly, no significant differences among all four subsets of IIM were detected (p>0.05 for all comparisons) **(** **Figure 1A** **)**.

### Hsp90 Plasma Levels in Relation to Disease-Related Features: A Cross-Sectional Analysis

Increased Hsp90 plasma levels were found in male patients and those with IIM-associated interstitial lung disease, cardiac involvement, dysphagia, the positivity of anti-Ro (52kDa) antibodies, and current use of conventional synthetic antirheumatic drugs (csDMARDs) (p<0.05 for all comparisons to patients without these features) (**Table 3** **).** Furthermore, the bivariate analysis demonstrated a significant association of plasma Hsp90 with several clinical and laboratory measures of disease activity and damage. A positive correlation of plasma Hsp90 was found with enzymes reflecting skeletal muscle involvement: lactate dehydrogenase (LD; strong correlation), and aspartate aminotransferase (AST; moderate correlation) (**Figures 1B, C**). In addition, increased plasma Hsp90 was weakly associated with elevated alanine aminotransferase (ALT), decreased muscle strength (decreased MMT-8 total score, specifically in the proximal muscles including m. biceps brachii, m. gluteus maximus, and m. iliopsoas), increased disease activity (MITAX, MYOACT, pulmonary and muscle disease activity, and global disease activity assessed by both the patient and the doctor), increased damage (MDI extent, severity, and extended), and higher current prednisone equivalent dose (**Supplementary Table 2**). Differences in significant disease-related predictors of plasma Hsp90 among individual subtypes of IIM are presented in **Table 4**. To validate the reliability of potential predictors of Hsp90 levels, which were established by the bivariate analysis, three main variables were selected for a multivariate regression analysis model: a composite of muscle enzymes (CK, LD, AST, and ALT), MITAX, and the current prednisone equivalent dose. However, only association with muscle enzymes remained significant (β=0.358, p<0.001) in this multivariate analysis model (**Supplementary Table 2**). Concentrations of extracellular Hsp90 were not significantly affected by other main clinical or laboratory parameters of IIM, such as disease duration (either from the first symptom or from the diagnosis), MSAs, or MAAs (except for anti-Ro-52) (data not shown).

**Table 3:** Elevated plasma Hsp90 in subgroups of patients with IIM.

Parameter (number of patients presented with the parameter)	Present median (IQR)	Absent median (IQR)	p-value
Male sex (n=79)	25.3 (15.6 – 50.0)	19.6 (13.4 – 35.6)	0.040
Interstitial lung disease (n=126)	25.4 (15.5 – 50.7)	18.9 (12.8 – 30.3)	0.003
Cardiac involvement (n=50)	27.5 (18.1 – 51.5)	19.3 (13.3 – 39.3)	0.004
Dysphagia (n=120)	25.0 (15.9 – 50.0)	18.2 (13.4 – 34.3)	0.018
Ro-52 (n=80)	29.6 (15.6 – 57.2)	19.2 (13.9 – 31.4)	0.001
csDMARDs (n=111)	22.0 (15.6 – 50.4)	18.9 (12.8 – 32.1)	0.014

csDMARDs, current treatment with conventional synthetic antirheumatic drugs; IIM, idiopathic inflammatory myopathies; IQR, interquartile range; Ro, anti-Ro52 autoantibodies (52 kDa, against cytoplasmic RNA and associated peptides).

**Table 4:** Significant disease-related predictors of Hsp90 plasma levels in patients with individual subsets of IIM based on bivariate correlations.

	(Spearman’s r; p-value)			
Parameter	PM (n=104)	DM (n=104)	CDM (n=42)	IMNM (n=27)
C-reactive protein			0.497; 0.007	
Lactate dehydrogenase	0.636; 0.001	0.574; 0.001	0.535; 0.004	0.584; 0.002
Creatine kinase				0.485; 0.014
Aspartate aminotransferase	0.485; 0.001	0.317; 0.001		0.475; 0.019
Alanine aminotransferase	0.234; 0.015		0.395; 0.042	
Manual Muscle Testing 8 (MMT-8) total		-0.210; 0.039		
Myosits intention to treat activity index (MITAX)	0.223; 0.024			0.504; 0.028
Patient disease global activity (PDGA)	0.258; 0.010	0.244; 0.016		
Doctor disease global activity (DGDA)	0.264; 0.008			0.474; 0.040
Pulmonary disease activity		0.232; 0.021		
Muscle disease activity	0.247; 0.012			
Constitutional disease activity	0.250; 0.011			
Myositis damage index (MDI) extent	0.248; 0.025	0.224; 0.049		
Myositis damage index (MDI) extended		0.270; 0.017		
Current prednisone equivalent dose	0.382; 0.001			0.450; 0.036


CDM, cancer-associated dermatomyositis; DM, dermatomyositis; IIM, idiopathic inflammatory myopathies; IMNM, immune-mediated necrotizing myopathy; PM, polymyositis.

In addition, weak to moderate positive correlations between Hsp90 and several crucial cytokines/chemokines involved in the pathogenesis of IIM were identified by the bivariate analysis, such as IL-1β, IL-6, IL-8, IL-17, interferon (IFN)-γ, C-X-C motif chemokine ligand 10 (CXCL10), monocyte chemoattractant protein 1 (MCP1, CCL2), macrophage inflammatory protein 1-alpha (MIP-1-α, CCL3), MIP-1-β (CCL4), vascular endothelial growth factor (VEGF), and platelet-derived growth factor (PDGF-BB) (p<0.05 for all correlations) (**Table 5** **)**.

**Table 5:** Cytokines and chemokines involved in the pathogenesis of IIM and their correlation to plasma Hsp90 based on the bivariate analysis in all IIM patients (n=277).

Cytokine/Chemokine	Spearman’s r	p-value
Interleukin 1β	0.188	0.002
Interleukin 6	0.182	0.003
Interleukin 8	0.242	<0.001
Interleukin 17	0.201	0.001
Interferon γ	0.229	<0.001
C-X-C motif chemokine ligand 10 (CXCL10)	0.224	0.001
Monocyte chemoattractant protein 1 (CCL2)	0.161	0.007
Macrophage inflammatory protein 1-α (CCL3)	0.208	<0.001
Macrophage inflammatory protein 1-β (CCL4)	0.229	<0.001
Vascular endothelial growth factor	0.260	<0.001
Platelet-derived growth factor-BB	0.426	<0.001

IIM, idiopathic inflammatory myopathies.

### The Impact of Pharmacotherapy on Plasma Hsp90: A Longitudinal Analysis (LC1 and LC2)

The potential effect of the standard-of-care pharmacotherapy was assessed in two independent cohorts of IIM patients with longitudinally collected blood samples: LC1 comprising treatment naïve patients (n=11) and patients with early disease (n=28) with recently started pharmacological treatment, and LC2 representing patients with established disease (n=23) with ongoing pharmacological treatment.

**Figure 2:**
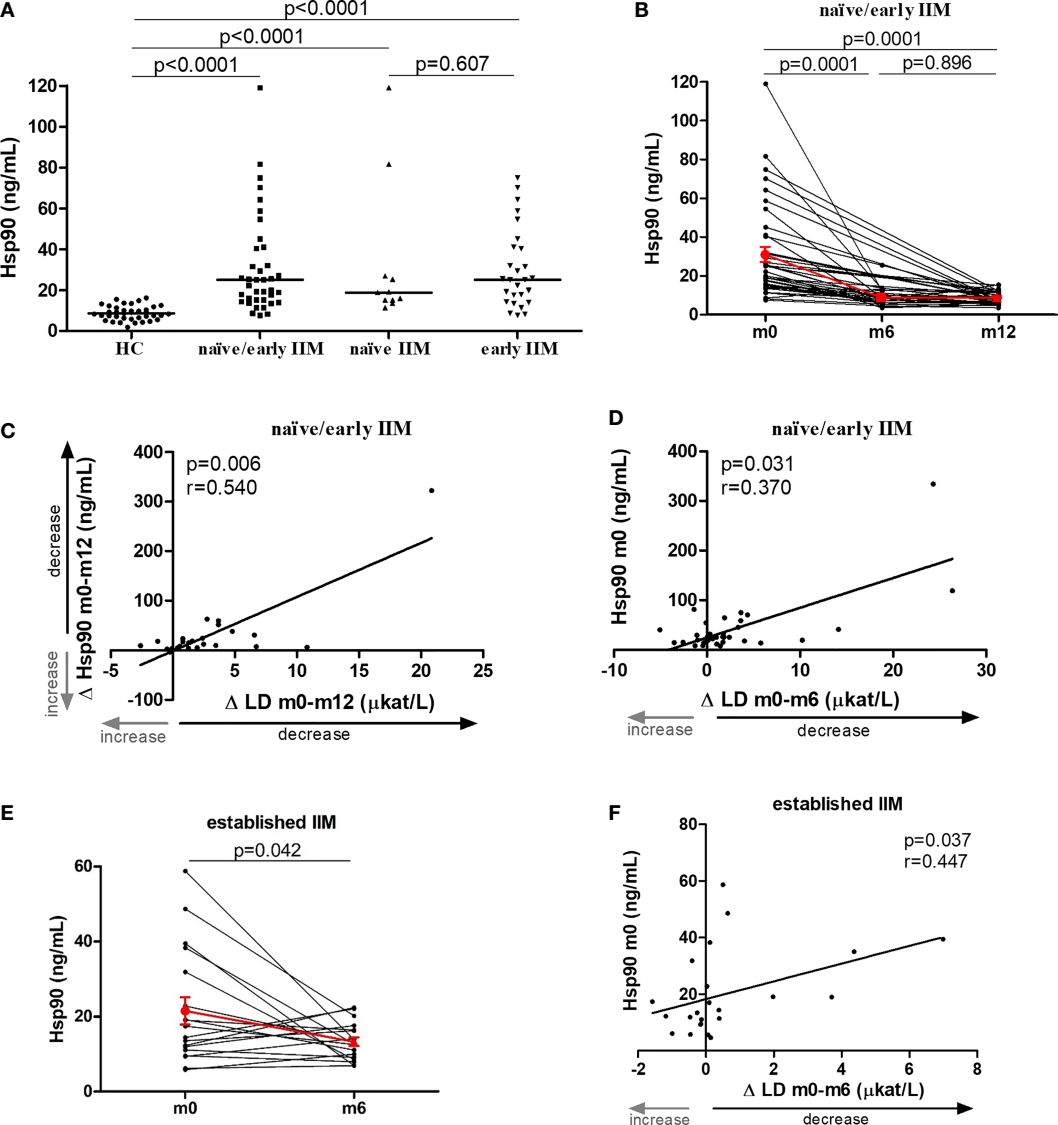
The effect of pharmacotherapy on plasma Hsp90 levels in patients with IIM. **(A)** Plasma Hsp90 levels were increased in both treatment naïve patients with idiopathic inflammatory myopathies (naïve IIM) and patients with short disease duration with recently started pharmacological treatment (early IIM) compared to healthy controls (HC). Horizontal bars represent the median. **(B)** Upon 6 and 12 months of standard-of-care pharmacotherapy, plasma Hsp90 levels significantly decreased in treatment naïve/early IIM patients. **(C)** The 12-month change in Hsp90 was associated with the 12-month change in LD levels. **(D)** Baseline Hsp90 predicted the 6-month change in LD levels. **(E)** In IIM patients with established disease, ongoing standard-of-care pharmacotherapy resulted in a significant decrease in plasma Hsp90 levels over six months. **(F)** Baseline Hsp90 levels were able to predict the 6-month change in the levels of LD. The red line and whiskers represent the mean and standard error of the mean **(B, E)**.

In LC1, significantly increased levels of plasma Hsp90 compared to age- and sex-matched healthy controls (n=39) were found in both treatment naïve patients, in patients with early disease, and combined (healthy controls: 8.6 [6.3–11.4], treatment naïve 18.9 [15.2–27.0], early disease 25.1 [14.3–41.1], treatment naïve/early disease 25.1 [15.2–41.1] ng/mL, p<0.0001 for all mentioned comparisons). No significant differences were observed between treatment naïve patients and patients with early disease (p=0.607) (**Figure 2A**). In brief, pharmacological treatment significantly decreased the mean serum levels of LD and CK at month 6 and 12 compared to baseline (LD: by 38% and 48%; CK: by 40% and 69%, respectively, p<0.01 for all comparisons) (**Table 2**). Upon treatment, we found a significant decrease in Hsp90 plasma levels in the whole LC1 at both month 6 and 12 compared to baseline levels (baseline: 25.1 [15.2–41.1], month 6: 7.8 [5.8–11.6], month 12: 7.7 [6.6–11.8] ng/mL, p=0.0001 for both comparisons) (**Figure 2B**). The decrease in plasma Hsp90 levels over 12 months was strongly associated with the decrease in LD over 12 months (r=0.540, p=0.006) (**Figure 2C**). Moreover, baseline Hsp90 was able to predict the change in LD levels at both 6 months (r=0.370, p=0.031) (**Figure 2D** **)** and 12 months (r=0.458, p=0.021).

In patients with established disease (LC2), we did not observe any significant change in the mean serum levels of LD and CK at month six compared to baseline (LD: -9%, p=0.897; CK: +5%, p=0.154) (**Supplementary Table 1**). However, upon treatment, we also detected a significant decrease in plasma Hsp90 levels at month six compared to baseline levels (baseline: 15.7 [9.8–29.5], month 6: 12.9 [9.7–16.6] ng/mL, p=0.042) (**Figure 2E**). In line with the findings in LC1, baseline Hsp90 in LC2 was able to predict the change in LD levels at month six (r=0.447, p=0.037) (**Figure 2F**). In addition, Hsp90 baseline levels also predicted the change in AST and CK levels at month six (r=0.494, p=0.020; r=0.430, p=0.046, respectively).

### Performance of Plasma Hsp90 in Comparison to Traditional Soluble Biomarkers

We evaluated the ability of plasma Hsp90 to distinguish IIM patients and its subsets (PM, DM, CDM and IMNM) from HC using the AUC-ROC analysis, and compared it to that of the traditional serum biomarkers such as CRP, CK and LD (**Supplemetary Table 3**). Plasma Hsp90 outperformed serum CRP and CK levels in discriminating between IIM vs. HC **(** **Supplementary Figure 1A** **)**, as well as between PM vs. HC **(** **Supplementary Figure 1B** **)** and DM vs. HC **(** **Supplementary Figure 1C** **)**, as demonstrated by higher AUC, sensitivity, specificity, and odds ratio (**Supplementary Table 3**). The discriminatory ability between CDM vs. HC **(** **Supplementary Figure 1D** **)**, and IMNM vs. HC **(** **Supplementary Figure 1E** **)** was comparable between Hsp90 and CK (**Supplementary Table 3**). Notably, out of all four biomarkers, plasma Hsp90 attained slightly higher AUC for discriminating between IIM patients with and without interstitial lung disease, and with and without cardiac involvement **(** **Supplementary Figures 1F-G** and **Supplementary Table 3**).

## Discussion

In the present study, we demonstrate elevated systemic levels of Hsp90 in patients with IIM and their association with several disease-related clinical and laboratory features. To date, several different roles of Hsp90 in muscle biology have been proposed. During myogenesis, increased expression of Hsp90 and ubiquitin in regenerating myofibers suggests that both are largely regulated by the activation of developmental mechanisms rather than by any particular disease (26). In damaged muscle fibers, De Paepe et al. propose a general protective role of Hsp90, i.e., counteracting mechanisms involved in the injury of myofibers. Whereas in PM or IBM, they suggest a specific cytotoxic role in the active invasion of myofibers by macrophages and cytotoxic T-cells, which is typically found in these subtypes of IIM (3, 17–19). Furthermore, Hsp90 alpha is secreted by activated endothelial cells and was postulated as a positive regulator of angiogenesis in wound healing (27). We hypothesize that damage to muscle fibers could be associated with an increased formation of a complex of Hsp90 with its client proteins and stabilizing peptides. However, these Hsp90-antigen complexes are recognized by antigen-presenting cells (APCs) such as macrophages or dendritic cells, and are presented by MHC class I or II molecules on APCs to CD8 or CD4 T lymphocytes, which actively invade non-necrotic muscle fibers or infiltrate the perivascular milieu (3, 17, 28). Activation of inflammatory cells leads to a release of pro-inflammatory cytokines and chemokines and an increased production of nitric oxide, which enhances the cytotoxicity of macrophages in myofibers (19, 28, 29).

Hsp are traditionally regarded as intracellular molecules. However, upon necrotic cell death and other stress stimuli, Hsp can be released into the extracellular compartment (29). In line with an increased Hsp90 expression in the involved skeletal muscles demonstrated by de Paepe et al. (17–19), herein, we demonstrate for the first time increased systemic levels of Hsp90 in IIM patients compared to healthy controls. Systemic levels of Hsp90 were comparable among all four IIM subtypes investigated (PM, DM, CDM, IMNM). Since Hsp90 is commonly overexpressed in cancer cells and secreted Hsp90 is believed to promote cancer cell invasion and metastasis (8, 30), it may be surprising that plasma Hsp90 was not actually higher in CDM, possibly due to the different volumes of the involved tissues. Increased plasma Hsp90 does not seem to be specific for IIM. Using the same methodology, our recently published studies demonstrated increased plasma Hsp90 also in patients with axial spondyloarthritis (axSpA) and SSc, and comparable levels in psoriatic arthritis (PsA) when compared to healthy individuals (15, 16). Furthermore, increased plasma Hsp90 in IIM patients was associated with several measures of disease activity (e.g., elevated serum levels of LD, AST and ALT, increased muscle weakness, higher physicians’ assessment of global, muscle and pulmonary activity, MITAX, MYOACT) and damage (increased MDI). However, all of these associations (except for that with LD) were weak. Higher plasma Hsp90 was detected in patients with interstitial lung disease (ILD) or anti-Ro-52 antibodies, which are associated with ILD in IIM (31) and cardiac involvement. These findings are in line with a possible role of Hsp90 previously described in SSc-ILD, idiopathic pulmonary fibrosis, heart failure and pathological cardiac remodeling (15, 32–34). Moreover, increased Hsp90 was found in patients with dysphagia and in those currently treated with csDMARDs or with a higher prednisone equivalent dose, which is in line with elevated Hsp90 in patients with higher disease activity. Higher Hsp90 in male IIM patients could be due to a larger muscle mass and higher disease activity in males than in females (MYOACT: 0.08 [0.015-0.135] vs 0.04 [0.02-0.11], respectively, p=0.082). In addition, elevated plasma Hsp90 was weakly associated with increased systemic levels of several key pro-inflammatory and pro-angiogenic cytokines/chemokines implicated in the pathogenesis of IIM, e.g., IL-1β, IL-6, IL-8, IL-17, IFN-γ, CXCL10, CCL2-4, VEGF, and PDGF (1–3, 35–42). However, only the muscle enzymes were confirmed by multivariate analysis as the strongest predictors of plasma Hsp90 in IIM.

Due to the well-known role of Hsp90 in regulating the activity of glucocorticoid receptors (43, 44), the fact that corticosteroids remain the cornerstone therapy in IIM (1, 2), and the positive correlation between plasma Hsp90 and the current prednisone equivalent dose in our cross-sectional cohort, we were interested in analyzing plasma Hsp90 in glucocorticoid naïve IIM patients and assessing the effect of glucocorticoid therapy (with csDMARDs). In treatment naïve IIM patients, plasma Hsp90 was higher than in healthy controls and was comparable to patients with short disease duration, who recently commenced pharmacological treatment (LC1). Interestingly, pharmacological treatment significantly decreased plasma Hsp90 in the pooled treatment naïve/early IIM group (LC1), which was associated with a decline in serum LD levels. A similar decrease in Hsp90 induced by pharmacological therapy was observed in patients with established IIM (LC2). Of particular interest, baseline Hsp90 levels were able to predict the 6-month change in serum LD levels in both LC1 and LC2. Similarly, our recently published study in patients with active SSc-ILD treated with cyclophosphamide demonstrated that baseline Hsp90 predicted the 12-month improvement in diffusing lung capacity for carbon monoxide (DLCO) (15).

Given the lack of organ-specific and disease-specific biomarkers for IIM used in routine clinical practice, we analyzed the diagnostic utility of plasma Hsp90 in IIM and its subsets and compared it to that of traditionally used soluble biomarkers such as CK, LD and CRP (45). Additionally, muscle-derived enzymes such as CK, aldolase, ALT, AST and LD have been used as indirect markers of various conditions causing myolysis, and usually indicate disease activity in patients with IIM (45). Due to their relative muscle specificity, serum CK levels are most frequently used for the diagnosis and monitoring of IIM, while the other markers (such as LD and transaminases) are present in almost all living cells and are far less specific for muscle damage (45). Moreover, elevated serum CRP is rarely seen in IIM, and is mostly observed in IIM-associated arthritis, ILD or cancer (46–48). In contrast, based on our diagnostic utility analysis, plasma Hsp90 outperformed CK and CRP in distinguishing IIM from HC, as well as PM and DM from HC. Of particular interest, plasma Hsp90 attained a slightly higher AUC than CK, LD or CRP in distinguishing IIM patients with ILD and cardiac involvement from those without these manifestations. The latter finding is of some potential, since CRP and ESR are the only routine biochemistry parameters demonstrated to be associated with PM/DM-ILD according to a recent meta-analysis (47). It is also worth mentioning that cardiac troponin isoform I (cTnI) is the only reliable serum marker associated with IIM-related myocardial damage, while other traditional cardiac enzymes need to be interpreted with caution (49, 50).

There are a few limitations to our study. Since IIM is an orphan disease, the number of patients for longitudinal analyses was relatively limited. However, possible selection bias was minimized by consecutive recruitment of patients. Neither aldolase, nor traditional serum markers of myocardial damage were available in our patients for comparative diagnostic utility analysis. Most of the detected associations of plasma Hsp90 with clinical or laboratory parameters of interest were weak and thus need to be interpreted with caution regarding their potential clinical utility. Furthermore, the results of disease activity (MITAX or MYOACT) were unavailable for most of the patients in the longitudinal cohorts at the follow-up examinations, which precluded us from evaluating the associations of the dynamics of plasma Hsp90 with disease activity. Despite these limitations, we believe that our study provides substantial evidence for plasma Hsp90 as a potential biomarker for IIM.

## Conclusion

In summary, this is the first study demonstrating elevated systemic levels of Hsp90 in patients with IIM and its potential association with several measures of disease activity and tissue damage, including functional impairment of the muscle and organ involvement. Plasma Hsp90 decreased upon pharmacological treatment in IIM patients with both early and established disease, and was able to predict the response to standard-of-care pharmacological therapy. Plasma Hsp90 demonstrated slightly superior ability to discriminate IIM patients from healthy controls, as well as IIM patients with interstitial lung disease or cardiac involvement from those without these manifestations, when compared to traditional soluble biomarkers such as CRP and CK. Thus, plasma Hsp90 could be a potential novel biomarker of disease activity, involvement of the skeletal muscle, lungs and heart, and a promising predictor of treatment response in IIM.

## Data Availability Statement

The original contributions presented in the study are included in the article/**Supplementary Material**. Further inquiries can be directed to the corresponding author.

## Ethics Statement

The studies involving human participants were reviewed and approved by Ethics Committee of the Institute of Rheumatology, Na Slupi 450/4, 12800 Prague 2, Czech Republic, with study reference number 5689/2015. The patients/participants provided their written informed consent to participate in this study.

## Author Contributions

HŠ, MŠ, KS, LŠ, JZ, JV, and MT designed the study. HŠ, SO, MŠ, BH, KB, OK, HM, KS, KP, LŠ, JZ, JV, and MT collected patient data. HŠ, MK, and MT performed the statistical analysis. HŠ and MT prepared the original draft of the manuscript. All authors critically interpreted the results, reviewed the draft version and approved the final manuscript.

## Funding

This work was supported by the Ministry of Health of the Czech Republic 023728, grant nr. 16-33542A, 16-33574A, NV18-01-00161A, SVV 260523, GAUK 312218 and BBMRI-CZ LM2018125.

## Conflict of Interest

The authors declare that the research was conducted in the absence of any commercial or financial relationships that could be construed as a potential conflict of interest.

## Publisher’s Note

All claims expressed in this article are solely those of the authors and do not necessarily represent those of their affiliated organizations, or those of the publisher, the editors and the reviewers. Any product that may be evaluated in this article, or claim that may be made by its manufacturer, is not guaranteed or endorsed by the publisher.

## Glossary

**Table:**

ALT	alanine aminotransferase
ANA	antinuclear antibodies
ANOVA	analysis of variance
anti-EJ	anti-glycyl-tRNA synthetase
anti-Jo-1	anti-histidyl-tRNA synthetase
anti-MDA5	anti-melanoma differentiation-associated gene 5
anti-Mi-2	antinuclear helicase 218/240 kDa
anti-NXP2	anti-nuclear matrix protein 2
anti-OJ	anti-isoleucyl-tRNA synthetase
anti-PL-12	anti-alanyl-tRNA synthetase
anti-PL-7	anti-threonyl-tRNA synthetase
anti-PM-Scl	anti-core complex 11-16 proteins
anti-SAE	anti-small ubiquitin-like modifier 1
anti-TIF1-γ	anti-transcriptional intermediary factor-1
AST	aspartate aminotransferase
AUC	area under the curve
ax-SpA	axial spondyloarthritis
CDM	cancer-associated dermatomyositis
CK	creatine phosphokinase
CRP	C-reactive protein
csDMARDs	conventional synthetic antirheumatic drugs
DM	dermatomyositis
ENMC	European Neuromuscular Centre
ER	endoplasmic reticulum
ESR	erythrocyte sedimentation rate
FGF	fibroblast growth factor
G-CSF	granulocyte colony-stimulating factor
GM-CSF	granulocyte-macrophage colony-stimulating factor
HAQ	health assessment questionnaire
Hsp	heat-shock protein
IBM	inclusion body myositis
IFN-γ	interferon-γ
IIM	idiopathic inflammatory myopathies
IL	interleukin
IMACS	International Myositis Assessment & Clinical Studies Group
IMNM	immune-mediated necrotizing myopathy
IoRP	Institute of Rheumatology in Prague
IP-10	interferon gamma-induced protein 10
IQR	interquartile range
LC	longitudinal cohort
LD	lactate dehydrogenase
MAAs	myositis-associated autoantibodies
Mb	myoglobin
MCP-1	monocyte chemoattractant protein 1
MDA	muscle disease activity
MDAAT	Myositis Disease Activity Assessment Tool
MDI	myositis damage index
MIP	macrophage inflammatory protein
MITAX	Myositis Intention to Treat Activity Index
MMT	manual muscle testing
MMT-8	manual muscle testing of 8 muscle groups
MSAs	myositis-specific autoantibodies
MYOACT	Myositis Disease Activity Assessment visual analogue scale
PDGF	platelet-derived growth factor
PM	polymyositis
RA	rheumatoid arthritis
RANTES	regulated on activation/normal T cell expressed and secreted
ROC	receiver operating characteristic
SLE	systemic lupus erythematosus
SSc	systemic sclerosis/scleroderma
TNF	tumor necrosis factor
TNF	tumor necrosis factor
VAS	visual analogue scale
VEGF	vascular endothelial growth factor
