# Supplementary material for: Hsp90 Levels in Idiopathic Inflammatory Myopathies and Their Association With Muscle Involvement and Disease Activity: A Cross-Sectional and Longitudinal Study
Source: Front Immunol. 2022 Jan 28;13:811045. doi: 10.3389/fimmu.2022.811045 (PMC8832010; doi:10.3389/fimmu.2022.811045)

**Supplementary material**

**Supplementary Table 1** Baseline clinical characteristics of IIM patients with established disease treated with standard-of-care pharmacological therapy: longitudinal cohort 2

| **Parameter** | **Baseline (Month 0)**  **(n = 23)** | **Month 6**  **(n = 23)** |
| --- | --- | --- |
| Sex: Female/Male, n (%) | 21 (91)/2 (9) |  |
| Age, years | 58.0 (52.0 – 65.0) |  |
| **Clinical features** |  |  |
| Disease duration, years | 2.8 (1.4 – 8.3) |  |
| IIM subtype, n (%): PM/DM/IMNM | 10 (44)/11 (48)/2 (8) |  |
| IIM-associated symptoms, n (%): MW/SR/MH/  RP/A/ILD/CI/D | 23 (100)/4 (17)/3 (13)/  7 (30)/1 (4)/7 (30)/5 (22)/7 (30) |  |
| MITAX | 0.2 (0.1 – 0.4) |  |
| MYOACT | 0.07 (0.03 – 0.13) |  |
| MDI extent | 0.1 (0.0 – 0.1) |  |
| MMT-8 | 64.0 (55.0 – 71.0) |  |
| HAQ | 1.3 (0.6 – 1.9) |  |
| **Laboratory features** |  |  |
| Autoantibodies, n (%):  ANA/Mi-2/TIF1/MDA5/  SAE/NXP2/SRP/HMGCR/Jo-1/  PM-Scl/snRNP/Ku/Ro-52/Ro-60 | 14 (61)/1 (4)/2 (9)/1 (4)/  1 (4)/1 (4)/2 (8)/0 (0)/7 (30)/  2 (9)/0 (0)/0 (0)/7 (30)/3(13) |  |
| CRP, mg/L | 4.1 (1.4 – 8.2) | 2-9 (1.4 – 10.4) |
| CK, μkat/L | 1.3 (0.8 – 3.9) | 1.7 (0.8 – 4.0) |
| LD, μkat/L | 3.7 (3.4 – 4.7) | 3.7 (3.2 – 4.9) |
| **Current treatment:**  Prednisone equivalent dose, mg/day  MTX/CPA/AZA/CSA/ LEF/MMF/SAS/HQ/TAC, n (%) | 15.0 (5.0-30.0)  7 (30)/1 (4)/2 (9)/2 (9)/  2 (9)/0 (0)/0 (0)/1 (4)/0 (0) | 7.5 (5.0-15.0)  6 (26)/1 (4)/5 (22)/2 (9)/  3 (13)/0 (0)/0 (0)/0 (0)/1 (4) |
| *Data are presented as median (inter-quartile range) unless stated otherwise;* *A, arthritis; ANA, antinuclear antibodies; AZA, azathioprine; CI, cardiac involvement; CK, creatine kinase; CPA, cyclophosphamide; CRP, C-reactive protein; CSA, cyclosporin A; D, dysphagia; DM, dermatomyositis; GC, glucocorticoids; HAQ, Health Assessment Questionnaire; HMGCR, anti-3-hydroxy-3-methylglutaryl-CoA reductase; HQ, hydroxychloroquine; IIM, idiopathic inflammatory myopathy; ILD, interstitial lung disease; IMNM, immune-mediated necrotizing myopathy; Jo-1, anti-histidyl-tRNA synthetase; Ku, anti-Ku (against the nuclear DNA-dependent protein kinase subunit); LD, lactate dehydrogenase; LEF, leflunomide; MDA5, anti-CADM-140 (melanoma differentiation-associated gene 5); MDI, Myositis Damage Index; MH, mechanic's hands; Mi-2, antinuclear helicase 218/240 kDa; MITAX, Myositis Intention to Treat Activity Index; MMF, mycophenolate mofetil;MMT-8, Manual Muscle Testing of eight muscles; MTX, methotrexate; MW, muscle weakness; MYOACT, Myositis Disease Activity Assessment visual analogue scale; NXP2, anti-NXP2 (nuclear matrix protein); PM, polymyositis; PM-Scl, anti-Pm-Scl (anti-core complex 11-16 proteins); Ro, anti-Ro (52/60 kDa, against cytoplasmic RNA and associated peptides); RP, Raynaud's phenomenon; SAE, anti-SUMO1 (small ubiquitin-like modifier 1) activating enzyme; SAS, sulphasalazine; snRNP, small nuclear ribonucleoprotein; SR, skin rash; SRP, anti-signal recognition particles; TAC, tacrolimus;TIF1, anti-TIF1 (transcription intermediary factor-1).* | | |

**Supplementary Table 2** Disease-related predictors of Hsp90 plasma levels in patients with IIM based on bivariate correlations and multivariate regression analysis

| **Bivariate correlations of Hsp90 with IIM-related features (n=277)** | | |
| --- | --- | --- |
| **Parameter** | **Spearman's r** | **p-value** |
| Alanine aminotransferase | 0.181 | **0.003** |
| Creatine kinase | 0.109 | 0.076 |
| MMT-8 total | -0.126 | **0.042** |
| MMT-8 neck flexors | 0.064 | 0.325 |
| MMT-8 m. deltoideus | -0.082 | 0.188 |
| MMT-8 m. biceps brachii | -0.125 | **0.043** |
| MMT-8 wrist extensors | -0.049 | 0.432 |
| MMT-8 m. quadriceps femoris | -0.038 | 0.545 |
| MMT-8 m. gluteus maximus | -0.159 | **0.011** |
| MMT-8 m. gluteus medius | -0.116 | 0.062 |
| MMT-8 ankle dorsiflexors | -0.066 | 0.289 |
| MMT m. triceps brachii | -0.126 | 0.278 |
| MMT m. iliopsoas | -0.143 | **0.023** |
| Myositis intention to treat activity index (MITAX) | 0.175 | **0.004** |
| Myositis disease activity assessment visual analogue scale (MYOACT) | 0.159 | **0.012** |
| Patient disease global activity (PDGA) | 0.223 | **<0.001** |
| Doctor disease global activity (DGDA) | 0.217 | **<0.001** |
| Pulmonary disease activity | 0.201 | **0.001** |
| Muscle disease activity | 0.146 | **0.018** |
| Myositis damage index (MDI) extent | 0.215 | **0.003** |
| Myositis damage index (MDI) severity | 0.150 | **0.041** |
| Myositis damage index (MDI) extended | 0.187 | **0.011** |
| Current prednisone equivalent dose | 0.183 | **0.006** |
| **Multivariate regression analysis predicting Hsp90 based on selected IIM-related features (n=277)** | | |
| **Parameter** | **b (95% CI)** | **p-value** |
| Muscle enzymes  Myositis intention to treat activity index (MITAX)  Current prednisone equivalent dose | 15.7 (9.5;22.0)  11.1 (-37.8;60.1)  0.1 (-0.2;0.3) | **0.001**  0.654  0.632 |
| *IIM, idiopathic inflammatory myopathies; MMT, Manual Muscle Testing; MMT-8, MMT of 8 muscles; Muscle enzymes, a composite of creatine kinase, lactate dehydrogenase, aspartate aminotransferase, and alanine aminotransferase; Statistically significant relationships (p<0.05) are marked in bold.* | | |

**Supplementary Table 3** Diagnostic utility analysis on Hsp90 plasma levels in idiopathic inflammatory myopathies and their subsets. The analyses were based on AUC-ROC values. Specificity and sensitivity are provided.

| **Group** |  |  |  |  |  | **Correct** |
| --- | --- | --- | --- | --- | --- | --- |
| Biomarker | **AUC (95% CI)** | **Cut-off** | **Sensitivity** | **Specificity** | **OR (95% CI)** | **classification** |
| **IIM vs. HC** |  |  |  |  |  |  |
| Hsp90 | 0.846 (0.808-0.884) | 11.945 | 0.83 | 0.70 | 1.90 (1.61-2.24) | 78.6% |
| CRP | 0.696 (0.643-0.748) | 2.525 | 0.50 | 0.79 | 1.31 (1.16-1.48) | 59.6% |
| CK | 0.610 (0.556-0.664) | 2.920 | 0.48 | 0.93 | 1.62 (1.29-2.02) | 63.3% |
| LD | 0.888 (0.857-0.919) | 3.495 | 0.76 | 0.96 | 2.27 (1.86-2.78) | 82.5% |
| **PM vs. HC** |  |  |  |  |  |  |
| Hsp90 | 0.853 (0.807-0.900) | 11.945 | 0.84 | 0.70 | 1.84 (1.52-2.23) | 76.3% |
| CRP | 0.675 (0.608-0.743) | 1.675 | 0.66 | 0.59 | 1.31 (1.16-1.48) | 64.2% |
| CK | 0.666 (0.587-0.746) | 2.930 | 0.54 | 0.93 | 1.62 (1.29-2.02) | 76.3% |
| LD | 0.884 (0.837-0.930) | 3.505 | 0.75 | 0.96 | 2.27 (1.86-2.78) | 86.7% |
| **DM vs. HC** |  |  |  |  |  |  |
| Hsp90 | 0.839 (0.787-0.892) | 16.611 | 0.68 | 0.87 | 1.75 (1.46-2.08) | 74.9% |
| CRP | 0.683 (0.615-0.751) | 1.905 | 0.63 | 0.64 | 1.31 (1.16-1.48) | 64.7% |
| CK | 0.454 (0.367-0.540) | 2.920 | 0.31 | 0.93 | 1.62 (1.29-2.02) | 66.8% |
| LD | 0.873 (0.822-0.924) | 3.605 | 0.72 | 0.98 | 2.27 (1.86-2.78) | 86.4% |
| **CDM vs. HC** |  |  |  |  |  |  |
| Hsp90 | 0.782 (0.684-0.880) | 16.611 | 0.56 | 0.87 | 1.63 (1.31-2.02) | 70.4% |
| CRP | 0.816 (0.720-0.913) | 2.525 | 0.78 | 0.79 | 1.31 (1.16-1.48) | 78.4% |
| CK | 0.744 (0.612-0.875) | 2.435 | 0.67 | 0.86 | 1.62 (1.29-2.02) | 87.0% |
| LD | 0.898 (0.807-0.989) | 3.290 | 0.85 | 0.89 | 2.27 (1.86-2.78) | 92.0% |
| **IMNM vs. HC** |  |  |  |  |  |  |
| Hsp90 | 0.882 (0.811-0.953) | 16.767 | 0.76 | 0.88 | 1.76 (1.36-2.27) | 73.8% |
| CRP | 0.703 (0.582-0.824) | 2.370 | 0.56 | 0.76 | 1.31 (1.16-1.48) | 74.4% |
| CK | 0.878 (0.752-1.000) | 3.465 | 0.88 | 0.96 | 1.62 (1.29-2.02) | 92.5% |
| LD | 0.970 (0.932-1.000) | 4.220 | 0.88 | 0.99 | 2.27 (1.86-2.78) | 94.4% |
| **ILD vs. no ILD** |  |  |  |  |  |  |
| Hsp90 | 0.616 (0.545-0.687) | 23.908 | 0.56 | 0.68 | 1.02 (0.99-1.04) | 55.0% |
| CRP | 0.555 (0.483-0.628) | 3.740 | 0.41 | 0.70 | 1.03 (1.00-1.06) | 55.0% |
| CK | 0.479 (0.406-0.552) | 0.245 | 0.99 | 0.05 | 1.00 (0.97-1.02) | 50.0% |
| LD | 0.517 (0.444-0.590) | 3.295 | 0.82 | 0.26 | 1.00 (0.98-1.03) | 52.5% |
| **CI vs. no CI** | | |  |  |  |  |
| Hsp90 | 0.624 (0.544-0.704) | 24.271 | 0.67 | 0.61 | 1.00 (0.98-1.03) | 34.1% |
| CRP | 0.564 (0.474-0.654) | 2.705 | 0.58 | 0.57 | 1.01 (0.99-1.04) | 55.0% |
| CK | 0.398 (0.312-0.483) | 0.200 | 1.00 | 0.01 | 0.97 (0.92-1.01) | 45.8% |
| LD | 0.534 (0.453-0.616) | 3.635 | 0.83 | 0.33 | 0.98 (0.95-1.02) | 38.6% |

*AUC, Area under receiver operator characteristics curve; CDM, cancer associated dermatomyositis; CI, cardiac involvement; CI, confidence interval; CK, creatine kinase; CRP, C-reactive protein; DM, dermatomyositis; HC, healthy controls; Hsp90, heat shock protein 90; IIM, idiopathic inflammatory myopathies; ILD, interstitial lung disease; IMNM, immune-mediated necrotizing myopathy; LD, lactate dehydrogenase; OR, odds ratio; PM, polymyositis.*

**Supplementary Figure 1** ROC analysis of plasma Hsp90 levels in **(A)** all idiopathic inflammatory patients (IIM) vs. healthy controls (HC), **(B)** polymyositis (PM) vs. HC, **(C)** dermatomyositis (DM) vs. HC, **(D)** cancer-associated DM (CDM) vs. HC, **(E)** immune-mediated necrotizing myopathy (IMNM) vs HC, and in IIM patients **(F)** with and without interstitial lung disease (ILD) and **(G)** with and without cardiac involvement (CI). ROC analysis was also performed on traditional soluble biomarkers such as C-reactive protein (CRP), creatine kinase (CK), and lactate dehydrogenase (LD).


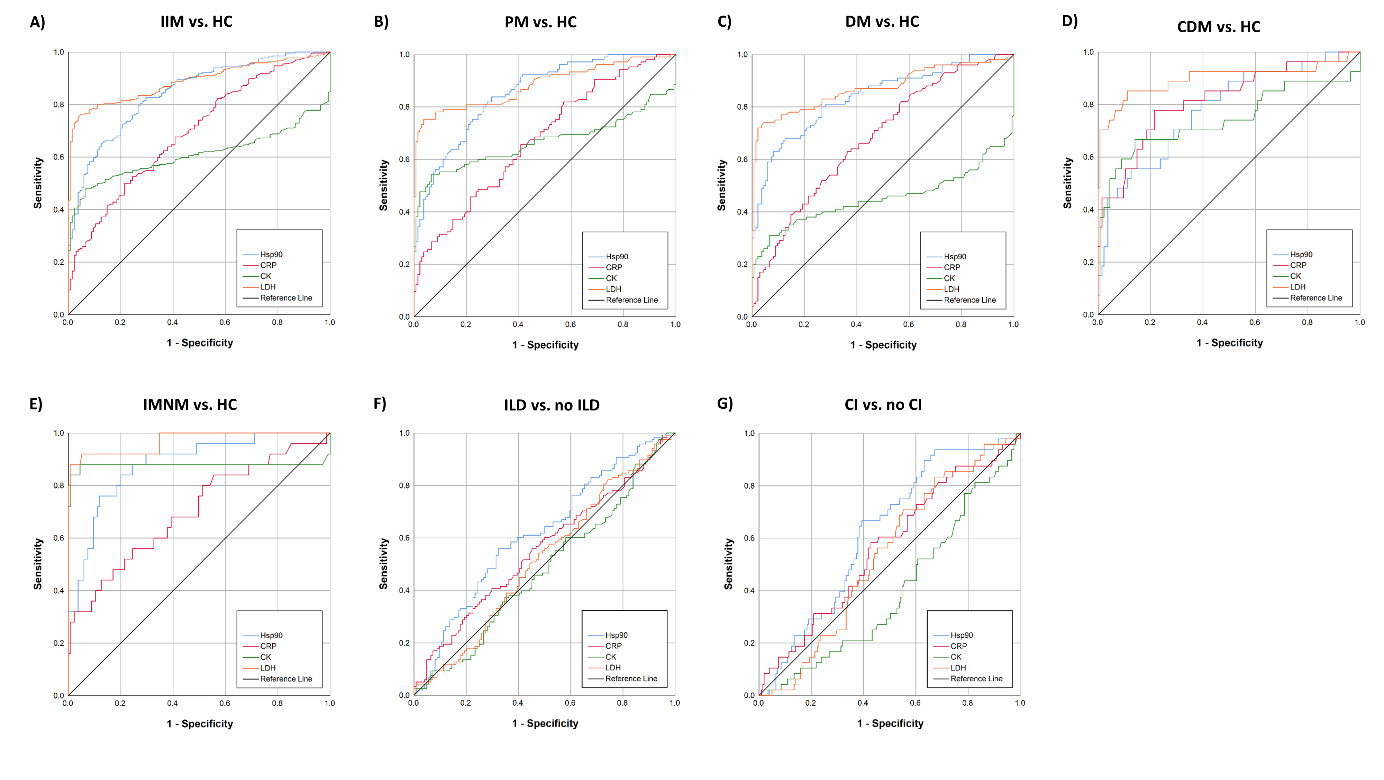

Supplement: Supplementary file 1 [file DataSheet_1.docx]
